# An efficiency assessment of digital technology empowering healthy aging in China: an empirical study of the “internet plus nursing services” pilot program based on the DEA model

**DOI:** 10.3389/fdgth.2026.1839189

**Published:** 2026-08-14

**Authors:** Lansong Huang, Anji Huang, Quansheng Wang

**Affiliations:** Discipline Inspection and Supervision School, Shandong University, Weihai, China

**Keywords:** blockchain, data envelopment analysis, digital technology, efficiency evaluation, healthy aging, smart wearables, telemedicine

## Abstract

This paper is rooted in the Chinese context and focuses on “the empowerment of health and older adults' care through digital technology” as its core topic. It aims to systematically evaluate the quantitative impact of digital technologies such as wearable devices and telemedicine on the efficiency and cost control of health and care services for older adults in China. This study adopts an empirical research approach that focuses primarily on quantitative analysis, supplemented by evidence from contextual case studies. First, through a contextual analysis of pilot cases involving “Internet Plus Nursing Services,” the study provides an in-depth examination of the practical application models and bottlenecks associated with the use of smart devices in monitoring older adults' activities of daily living (ADL), thereby providing a practical basis for the development of quantitative indicators. Second, using SPSS multi-option analysis, it quantifies the digital needs of various types of older adult care services, including home, community, and institutional care, and constructs a Data Envelopment Analysis (DEA) model to measure technological input and output efficiency. Empirical results show that applying digital technology can, on average, improve service response efficiency by approximately 25% and reduce operating costs by 15%–20% in specific scenarios. Still, bottlenecks such as “data silos” and insufficient age-friendliness exist. Finally, it prospectively analyses the development prospects of blockchain technology, ensuring secure, trusted data circulation and AI-driven, personalized health management, and proposes a transformation path of “technology integration—model innovation—institutional synergy.” This study provides empirical evidence and decision-making guidance to optimize resource allocation for digital care for older adults and improve overall service quality.

## 1 Introduction

As China's population aging continues to deepen, by 2024, the population aged 60 and older will exceed 280 million, accounting for over 20% of the total population, marking its entry into a deeply aging society. Increasingly, the number of very old, disabled, and solitary older adults is rising, leading to diversified, personalized, and refined demands for healthy aging services (1). Traditional healthy aging models, based primarily on offline nursing and institutional care, suffer from drawbacks such as low service efficiency, uneven resource allocation, high costs, and ineffective responses, making it difficult to meet the growing needs of older adults for healthy aging (2).

The rapid development of digital technology provides new opportunities to transform the healthy aging industry. The integration of technologies such as smart wearables, telemedicine, big data analytics, blockchain, and artificial intelligence with the healthy aging sector is deepening, gradually building an integrated “monitoring-assessment-intervention-nursing” service system. Smart wearables can collect real-time data on vital signs and activities of daily living for older adults, enabling early detection and intervention for health risks. Telemedicine breaks down spatial and temporal barriers, allowing older adults to enjoy quality medical services without leaving home, thereby reducing medical costs. Big data technology can integrate information on demand for and resources for older adult care services, optimizing resource allocation efficiency (3, 4).

From a theoretical perspective, the empowerment of health and care for older adults by digital technology has a deep theoretical foundation (5, 6). The theory of technological empowerment emphasizes that technology breaks development bottlenecks and optimizes existing processes by granting new capabilities to industries (7); the resource allocation theory suggests that scarce resources' supply-demand matching and utilization efficiency must be achieved through scientifically configured strategies (8); the service quality theory focuses on meeting service recipients' demands by optimizing service processes and response speeds (9). The integration of digital technology into health and care for older adults reflects these three main theories in practice: empowering service processes through smart devices and telemedicine, optimizing the allocation of medical and nursing resources, and improving the speed and personalization of service responses (10). However, although academia has focused on the application of digital technologies in older adults' care, existing research still has noticeable shortcomings: first, most studies emphasize qualitative descriptions or single technology analyses, lacking systematic quantitative evaluations of multitechnology integration and multiservice scenarios, especially quantitative research on the application effects of smart devices in monitoring activities of daily living (ADL); second, efficiency evaluation methods are not well developed, lacking scientific efficiency evaluation models such as Data Envelopment Analysis (DEA), making it difficult to accurately measure the actual impact of digital technologies on service efficiency and cost control; third, research on transformation pathways often remains at the macro level, with insufficient in-depth analysis of how emerging technologies like blockchain and artificial intelligence can address bottlenecks such as data silos and inadequate personalization.

To promote the digital transformation of health and older adults care, China has successively introduced policies such as the “14th Five-Year National Health Plan” and the “Digital Health Action Plan,” clearly stating the need to promote the deep integration of digital technology and health and older adults care, support the pilot construction of “Internet + Nursing Services,” and improve digital infrastructure for health and older adults care (8). In this context, based on the theoretical background and research shortcomings mentioned above, this study explores transformation pathways for empowering health and older adults' care through digital technology, quantitatively evaluates its impact on service efficiency and cost control, addresses current prominent issues in application, and anticipates future technological application prospects, which holds significant practical significance and theoretical value for promoting the high-quality development of the health and older adults' care industry and responding to the challenges of population aging.

## 2 Literature review and its theoretical basis

### 2.1 Literature review

Regarding the effectiveness of digital technologies (i.e., various technologies used to collect, store, transmit, analyze, and process data, with a particular focus on those deeply integrated into the health and older adult care sector, primarily including smart wearable technology, telemedicine, big data analytics, blockchain, and artificial intelligence) in the health and older adult care sector, existing research has primarily examined the practical benefits of technologies such as smart wearable devices and telemedicine (9). Research indicates that smart wearable devices can effectively monitor the risk of falls among older adults, reducing emergency response times by more than 50%; In particular, regarding the monitoring of Activities of Daily Living (ADL—the ability of older adults to independently perform daily activities such as eating, dressing, personal hygiene, toileting, walking, and climbing stairs), smart devices collect data in real time to analyze activity patterns and physical condition, promptly detecting abnormalities such as falls or sudden decreases in activity levels, thereby providing a crucial basis for health risk intervention and care services (10, 11). In addition, telemedicine can reduce the number of nonessential outpatient visits, lower healthcare costs by 25%–30%, and improve access to medical care. Despite the ongoing expansion of application scenarios, significant urban-rural and regional disparities persist in the adoption of digital technologies. While urban areas have relatively mature applications, rural areas face challenges such as weak infrastructure and low adoption rates (12, 13).

Health-centered older adult care refers to an approach that centers on the health needs of older adults and integrates various resources—including medical care, nursing, rehabilitation, health management, and older adult care services—to provide them with integrated services ranging from health monitoring, disease prevention, diagnosis, treatment, and nursing to rehabilitation, long-term care, and end-of-life care. It encompasses three primary models—home-based care, institutional care, and community-based care—and emphasizes the deep integration of “health” and “older adult care,” distinguishing it from traditional, single-dimensional models of older adult care. In terms of service model innovation, the integration of digital technology with older adult care resources has given rise to diversified health and older adult care service models. For example, the “remote home-based older adult care model” and the “smart community older adult care model” integrate digital technology with older adult care resources to integrate health monitoring, nursing services, and emergency response (14, 15). Relevant research has focused on exploring the feasibility and optimization pathways of these models. At the same time, application scenarios such as “Internet Plus nursing services” are continually expanding, providing a practical foundation for integrated services across scenarios (16, 17).

In response to the practical challenges facing the use of digital technology to enhance health and older adult care, scholars generally agree that there are currently four major issues: First, uneven digital infrastructure, with low coverage in rural and remote areas (18); second, low digital literacy among the elderly, making it difficult for them to use digital devices proficiently (19); third, insufficient data security and privacy protection, posing risks to the sharing of health data (20); and fourth, a disconnect between technology applications and the needs of older people, resulting in a lack of personalized services (21). In exploring pathways for transformation, existing research has proposed measures such as strengthening infrastructure, conducting digital literacy training, improving data security systems, and promoting the integration of technology and services. However, most studies lack empirical support, and their relevance and practicality need to be enhanced (19, 22).

In the areas of data security and cutting-edge technological innovation, scholars are increasingly focusing on the application of blockchain technology in the storage and sharing of health data, exploring how its decentralized and tamper-proof characteristics can address issues such as health data privacy breaches and “data silos.” At the same time, research into AI-driven personalized health management continues to deepen, and big data-based health assessments and personalized intervention plans have gradually been implemented, emerging as a key avenue for addressing the limitations of one-size-fits-all services (23, 24).

Based on a comprehensive review of the current state of research, the use of digital technology to empower healthy aging and older adult care—that is, the deep integration of digital technology with the healthy aging and older adult care sector to optimize service processes, integrate service resources, and innovate service models through digital technology, thereby achieving a digital, intelligent, and refined transformation of these services to enhance efficiency, reduce costs, and meet the diverse needs of older adults—has become a hot research topic. While some progress has been made in areas such as technology application, service models, and problem analysis, significant shortcomings remain: First, some research conclusions and service models lack targeted empirical analysis and quantitative evaluation; second, existing research largely focuses on a single technology or a single scenario, lacking systematic analysis of multi-technology integration and multi-service scenarios—in particular, there is a scarcity of quantitative studies on the effectiveness of smart devices in monitoring Activities of Daily Living (ADL); third, efficiency assessment methods are not sufficiently scientific, and the absence of a comprehensive efficiency evaluation model makes it difficult to accurately quantify the impact of digital technologies on service efficiency and cost control; Fourth, the transformation pathways lack practicality, and the analysis of the application prospects for emerging technologies such as blockchain and AI is not sufficiently in-depth.

### 2.2 Theoretical basis

#### 2.2.1 The theory of technological empowerment: the internal logic of digital technology reshaping older adults' care service capabilities

The traditional model of care for older adults and the sick is constrained by spatial and temporal barriers and the reliance on human labor, leading to inherent efficiency bottlenecks. The theory of technological empowerment transcends the limitations of the “technological tool theory,” emphasizing technology as an intrinsic driver of the reconstruction of capabilities and processes in industrial systems (25). In the context of health and older adults' care, the intervention of digital technologies, such as smart wearables and telemedicine, is not just an online substitution for offline services; rather, it grants the older adults' care system sensitivity and responsiveness beyond traditional human limitations through real-time data collection and algorithmic intervention, achieving a paradigm shift from “passive response” to “active prevention” (26). This article takes this theory as the logical starting point for analyzing how digital technologies can break the efficiency lock-in in traditional care for older adults, providing an ontological basis for deconstructing the enabling mechanism that transforms technological input into service efficiency.

#### 2.2.2 Resource allocation theory: an explanatory framework for data-driven optimal allocation of older adults' care resources

The scarcity and uneven distribution of health and care resources for older adults are the main constraints on the industry's development. Resource allocation theory focuses on the flow of resources in a multidimensional space and the matching of supply and demand. In the digital age, data has become a key driver of resource allocation optimization (27, 28). Digital technology reduces information asymmetry between medical care institutions, communities, and families, allowing precise scheduling and dynamic reorganization of physical resources, such as nursing staff and medical equipment, based on data flow, thus breaking the “island effect.” (29) This study, based on this theoretical perspective, regards the essence of digital empowerment as the optimization and reorganization of physical older adults' care resources by data elements, providing theoretical support for constructing logical connections between input and output elements in the DEA efficiency evaluation model and exploring ways to overcome imbalances in resource allocation.

#### 2.2.3 Service quality theory: evaluation benchmarks and evolutionary directions for heterogeneous demand adaptation

The ultimate goal of digital technology empowerment is to enhance the sense of gain and satisfaction among the older adult population, rather than merely pursuing technological iterations. Service quality theory emphasizes the matching of supply and demand and the perceived utility of service recipients, revealing that technological empowerment must adhere to the value principle of “people-oriented” (30). In the context of digital care for older adults, the concept of service quality has expanded to encompass responsiveness, reliability, and the personalization of digital systems. If technological supply fails to align with the real needs and digital literacy of older adults, it can easily lead to “technological disconnection” and resistance (31). This paper, based on this theory, incorporates demand heterogeneity and satisfaction perceptions into the output dimension of efficiency assessment, providing value criteria for examining existing shortcomings in the application of age-friendly technology and for developing advanced pathways to transform AI-driven personalized services.

## 3 Research design: methods, cases, and data

### 3.1 Overall research framework: An empirical approach focusing on quantitative methods and supplemented by contextual case studies

Given that the core objective of this study is to quantitatively measure the efficiency and costs of using digital technologies to enhance health and elderly care, the methodology employs an empirical research approach centered on quantitative analysis and supported by contextual case evidence. Specifically, the study first conducts a contextual analysis of pilot cases involving “Internet Plus Nursing Services” to provide a practical basis for analyzing bottlenecks in the application of digital technologies and for subsequently constructing a quantitative indicator system; Subsequently, large-sample questionnaire surveys and SPSS multiple-choice analysis were used to quantify demand, and Data Envelopment Analysis (DEA) and Tobit regression models were constructed to measure the efficiency of technological inputs and outputs, as well as their influencing factors; finally, quantitative results were integrated with case scenarios to identify trends and project transformation pathways. The contextual case studies are primarily used here to provide practical support for variable selection and the interpretation of results in the quantitative models, rather than as independent qualitative leads for analysis.

### 3.2 Case selection and contextual analysis: “internet plus nursing services” pilot program

City A, a national pilot city for the “Internet plus Nursing Services” initiative, was selected as a case study. The city has rolled out an ADL monitoring system—integrating smart wristbands, sleep monitoring pads, and emergency call devices—in multiple communities, serving more than 2,000 elderly individuals living alone. It has accumulated over 18 months of operational data, making it representative and ensuring data availability. This study utilizes the operational data and field survey records from this pilot program as contextual evidence, providing concrete real-world support for the definition of indicators (such as the average daily number of effectively handled abnormal events) and the interpretation of results in the quantitative assessment that follows.

### 3.3 Data sources and sample characteristics

Quantitative data were collected from a 2023 questionnaire survey of older adults in City A and its surrounding areas, as well as operational data from pilot institutions. A total of 1,200 questionnaires were distributed, and 1,028 were collected, yielding an effective response rate of 85.7%. The basic characteristics of the sample are shown in Table 1.

**Table 1:** Description of the basic characteristics of the survey samples (*N* = 1,028).

Characteristic variables	Category	Frequency	Percentage
Age	60–74 years	612	59.5
	75–84 years	328	31.9
	85 years and over	88	8.6
Gender	Male	496	48.2
	Female	532	51.8
Living arrangements	Living alone	187	18.2
	Living with a spouse	535	52.0
	Living with children	306	29.8
Preferred older adult care models	Home-based care	743	72.3
	Community-based care	205	19.9
	Institutional care	80	7.8
Chronic disease Prevalence	Having one or more types	821	79.9
	None	207	20.1
Digital device usage experience	Frequent Use	385	37.5
	Occasional Use	402	39.1
	Never used	241	23.4

### 3.4 Core research methods: SPSS multiple response analysis and DEA efficiency evaluation model construction

SPSS Multiple Response Analysis: For the question “Which digital eldercare services do you currently use or wish to use?” (multiple-choice question), the “Multiple Response Sets” function in SPSS is used to conduct frequency analysis and cross-tabulation analysis, clearly depicting the combination patterns of needs among different demographic groups.

DEA Efficiency Evaluation Model Construction: A total of 52 typical service units were selected from the expanded screening in the pilot area, including 22 community sites, 20 home service teams, 6 large-scale medical care institutions, and 4 regional remote nursing service centers, which serve as decision-making units (DMUs). Based on the original pilot in City A, data from 3 additional nursing pilot counties + the Internet in the vicinity were added. Invalid samples with missing data or with operations lasting less than 6 months were excluded, leaving 52 effective decision-making units. This greatly expands the sample size for DEA calculations, meeting statistical norms and the empirical reliability requirements for large-sample DEA modeling.

Input Indicators (X): Digital technology input cost (10,000 CNY), number of professional nursing staff (persons), and investment in traditional equipment (10,000 CNY).

Output Indicators (Y): Service coverage demand satisfaction weighted and calculated based on multiple-choice analysis (%) and the average daily number of effectively handled abnormal events (cases) and user satisfaction score (out of 100).

Using an input-oriented BCC model, we calculate overall technical efficiency (TE), pure technical efficiency (PTE), and scale efficiency (SE).

## 4 Analysis of the current status of digital technology empowerment and ADL monitoring application cases

### 4.1 Main modes of application of smart wearables and telemedicine in older adults' care

Current applications mainly focus on four major scenarios: safety monitoring (anti-wandering, fall detection), health management (chronic disease indicator monitoring, medication reminders), convenient communication (one-button call, video contact), and spiritual comfort (smart companionship). However, the level of integration among various technologies is low, and the data standards vary.

### 4.2 In-depth case analysis: application, effectiveness, and challenges of smart devices in ADL monitoring

As a contextual case study for this research, in the pilot program in City A, the system establishes behavioral baselines by monitoring data on ADLs, such as older adults' time out of bed, toilet frequency, and indoor activity trajectories, alongside algorithmic models. Once an anomaly is detected (e.g., prolonged inactivity or frequent nighttime awakenings), the system automatically alerts the platform and the family members' mobile phones.

Effectiveness: During the pilot period, a total of 127 fall risk events were effectively prewarned, 43 cases of potential health deterioration (e.g., frequent urination due to urinary tract infections) were identified, and the average emergency response time was reduced from 30 min for traditional phone calls for help to 12 min. The anxiety of family members was significantly reduced.

Challenges: 1) The data false alarm rate is approximately 15%, mainly due to changes in older adults' habits or improper device wearing; 2) older adults' resistance, feeling “monitored”; 3) Insufficient data literacy among caregivers, making it difficult to interpret deeper health information from the data; 4) Data are not interoperable with medical systems, so warning information cannot directly form electronic medical records or doctor's orders.

The outcomes and challenges revealed by the above case studies provide a practical basis and contextual support for setting the output indicators (such as the average daily number of effectively handled abnormal incidents) for the subsequent DEA efficiency assessment, as well as for selecting the influencing factors (such as digital skills training for nursing staff and data platform interoperability) in the Tobit regression model.

### 4.3 Heterogeneous service demand profile based on multi-option analysis

The results of the multi-option analysis of 1,028 questionnaires (see Table 2) show that the digital service demands of older adults exhibit clear diversification and structural differences. “Health monitoring and early warning” is a common core demand (71.2%), but different groups have different priorities: the older adult and those living alone have an urgent need for “emergency calls and safety monitoring” (58.3%); patients with chronic diseases are highly concerned with “remote medication guidance and consultations” (65.4%); while younger, more educated individuals prefer “online entertainment and social interaction” (44.1%). This provides direct evidence for the development of precise and differentiated technology empowerment strategies.

**Table 2:** Multi-option analysis of the demand for the digital older adult care service (*N* = 1,028, total responses = 2,878).

Demand category	Number of responses (*N*)	Response percentage	Case percentage
Health monitoring and early warning (e.g., blood pressure, heart rate monitoring)	732	25.4%	71.2%
Emergency call and safety monitoring (e.g., fall detection)	598	20.8%	58.2%
Remote medication guidance and online consultation	517	18.0%	50.3%
Life assistance and reminders (e.g., medication, activity reminders)	463	16.1%	45.0%
Online entertainment, learning, and socializing	314	10.9%	30.5%
Smart home control (e.g., lights, air conditioning)	254	8.8%	24.7%
Total	2,878	100.0%	280.0%

## 5 Evaluation of digital technology empowering healthy aging

### 5.1 Construction of efficiency evaluation indicator system

Based on the principles of scientificity, accessibility, and representativeness, and referencing the existing literature on the measurement of the efficiency of digital older adults' care services (such as the classification of input-output dimensions for smart older adults' care), combined with previous theories of technological empowerment and resource allocation, an input-output indicator system was constructed as shown in Table 3. In terms of input indicators, based on resource allocation theory, the investment cost of digital technology (X1), the number of professional caregivers (X2), and the input of traditional equipment and services (X3) were selected to reflect the core elements of capital and labor; In terms of output indicators, based on service quality theory and the logic of technological empowerment, we selected the service coverage satisfaction rate (Y1, where the calculation weight is derived from the earlier SPSS multi-option analysis of demand frequency), the daily average number of effectively handled abnormal incidents (Y2) reflecting the core effectiveness of technological empowerment, and comprehensive user satisfaction ratings (Y3) reflecting the perceived utility of service recipients.

**Table 3:** Efficiency evaluation indicator system for digital technology empowering health and older adult care services.

Indicator type	Indicator name	Indicator description and unit
Input indicators	X1: Digital Technology Input Cost	Annual total investment in smart devices, software platforms, data traffic, etc. (10,000 CNY)
	X2: Number of Professional Caregivers	Number of qualified full-time caregivers (persons)
	X3: Investment in Traditional Equipment and Services	Annual investment in non-digital equipment, facilities, transportation, etc. (10,000 RMB)
Output indicators	Y1: Service Coverage Demand Satisfaction	(Number of actual service items/Average number of demanded items for the target population of this unit derived from multi-option analysis) * 100%
	Y2: Daily Average Number of Effectively Handled Abnormal Events	Daily average number of health and safety incidents discovered and successfully handled through digital systems (incidents)
	Y3: User Comprehensive Satisfaction Score	Average satisfaction score obtained through a questionnaire survey (0–100 points)

### 5.2 Results of the ST measurement based on the DEA model

Using DEAP 2.1 software for calculation, the statistical results of the efficiency values for the 52 DMUs showed that the average overall technical efficiency (TE) was 0.842, which is close to the original small sample calculation of 0.845, indicating that the calculation results are robust after sample expansion, with an overall efficiency improvement potential of 15.8% remaining (Table 4).

**Table 4:** Internet + nursing services DEA efficiency values measurement results.

DMUID	Decision-making unit type	Comprehensive technical efficiency (TE)	Pure technical efficiency (PTE)	Scale efficiency (SE)	Returns to scale status
DMU1	Digital Transformation Department of Large-scale Older Adult Care Institutions	0.782	0.850	0.920	drs (decrement)
DMU2	Community Older Adult Care Service Center A Station	0.856	0.890	0.962	irs (Incremental)
DMU3	Medical and Older Adult Care Integration Institution Digital Platform	0.910	0.950	0.958	(Unchanged)
DMU4	Smart Older Adult Care Demonstration Community	1.000	1.000	1.000	(Unchanged)
DMU5	Home-based older adult care Service Team Group A	0.735	0.820	0.896	irs
DMU6	Regional Older Adult Care Information Center	0.892	0.930	0.959	drs
DMU7	Community Older Adult Care Service Center B Station	0.823	0.880	0.935	irs
DMU8	Home-based older adult care Service Team Group B	0.768	0.830	0.925	irs
DMU9	Medium-sized older adult care institution Smart Branch	0.881	0.910	0.968	-
DMU10	Telemedicine Service Center	0.942	0.970	0.971	drs
DMU11	Health Management Company Service Point	0.815	0.860	0.948	irs
DMU12	Digital-first Care Team	1.000	1.000	1.000	(Unchanged)
DMU13	Community Older Adult Care Service Center C Station	0.794	0.840	0.945	irs
DMU14	Home-based older adult care Service Team C Group	0.721	0.810	0.890	irs
DMU15	High-end Smart Older Adult Living Apartments	1.000	1.000	1.000	(Unchanged)
DMU16	Public Hospital Geriatrics Digital Project	0.863	0.920	0.938	drs
DMU17	Home-based older adult care Service Team D Group	0.689	0.790	0.872	irs
DMU18	Community Older Adult Care Service Center D Station	0.832	0.885	0.940	irs
DMU19	Social Welfare Institute Digital Upgrade Department	0.877	0.900	0.974	drs
DMU20	Home-based elder care service team E Group	0.751	0.820	0.916	irs
DMU21	Community Senior Care E-Station in Neighboring City	0.817	0.872	0.937	irs
DMU22	Community Older Adults Care Station F in a Neighboring City	0.843	0.896	0.941	irs
DMU23	County Home Care Group F	0.716	0.803	0.892	irs
DMU24	County Home Care Group G	0.742	0.819	0.906	irs
DMU25	County Medium-sized Medical and Nursing Branch	0.895	0.934	0.958	drs
DMU26	County Remote Nursing Substation 1	0.927	0.959	0.967	drs
DMU27	County Benchmark Smart Community Station	1.000	1.000	1.000	- (Unchanged)
DMU28	County Home Care Group H	0.708	0.795	0.891	irs
DMU29	Private Older Adults Care Institution 1 in Neighboring City	0.806	0.857	0.940	irs
DMU30	Private Older Adults Care Institution 2 in a Neighboring City	0.829	0.879	0.943	irs
DMU31	County Community Older Adults Care Station G	0.775	0.833	0.930	irs
DMU32	County Community Older Adults Care Station H	0.799	0.849	0.941	irs
DMU33	Cross-Regional Medical and Older Adults Care Outpatient 1	0.904	0.946	0.956	drs
DMU34	Cross-Regional Medical and Older Adults Care Outpatient 2	0.886	0.929	0.954	drs
DMU35	Home Older Adults Care Service Group I	0.697	0.784	0.889	irs
DMU36	Home Older Adults Care Service Group J	0.733	0.812	0.903	irs
DMU37	District County Remote Nursing Substation 2	0.915	0.952	0.961	drs
DMU38	District County Health Management Service Point 2	0.827	0.874	0.946	irs
DMU39	County High-End Smart Health and Older Adults Care Community	1.000	1.000	1.000	- (Unchanged)
DMU40	Community Older Adults Care Station I	0.811	0.865	0.937	irs
DMU41	Community Older Adults Care Station J	0.837	0.888	0.942	irs
DMU42	Digital Division of County Welfare Institutions	0.869	0.895	0.971	drs
DMU43	Home-based Older Adults Care Group K	0.725	0.808	0.897	irs
DMU44	Home-based Older Adults Care Group L	0.747	0.825	0.905	irs
DMU45	Comprehensive Older Adults Care Service Complex 1	0.899	0.939	0.957	drs
DMU46	Comprehensive Older Adults Care Service Complex 2	0.907	0.947	0.958	drs
DMU47	Community Health and Older Adults Care Service Station	0.788	0.841	0.937	irs
DMU48	Cross-district Benchmark Digital Nursing Center	1.000	1.000	1.000	- (Unchanged)
DMU49	County Community Older Adults Care Station K	0.769	0.827	0.930	irs
DMU50	Private Small-scale Smart Older Adults Care Station 1	0.802	0.853	0.940	irs
DMU51	Private Small-scale Smart Older Adults Care Station 2	0.819	0.871	0.940	irs
DMU52	Township Remote Consultation Service Point	0.872	0.901	0.968	drs

The average pure technical efficiency (PTE) was 0.903, which is higher than the average scale efficiency (SE) of 0.935, indicating that insufficient levels of management and technical application capabilities remain the main sources of inefficiency, rather than the scale of operations.

Among them, DMU4, DMU12, DMU15, DMU27, DMU39, and DMU48 reached the DEA effectiveness (TE = 1). The common characteristic of these units is a high degree of integration of digital technology into nursing processes, along with proper personnel training. On the contrary, units with lower efficiency commonly face problems such as low equipment utilization or insufficient data usage.

From the complete efficiency distribution, it can be seen that only 6 out of the 52 decision units achieved DEA efficiency, with platform services significantly more efficient overall compared to home services; a total of 29 units are in a state of increasing returns to scale, accounting for 55.8% of the total sample, still concentrated in the community and home service sectors. This suggests that at the current stage of development, moderate expansion in the scale of these units may lead to further efficiency improvements.

### 5.3 Analysis of efficiency influencing factors: a technological, human resources, and institutional perspective

To further explore the causes of the differences in DEA efficiency values, this paper combines the practical pain points identified in the previous case analysis (such as data interoperability issues and insufficient staff qualifications) with the four prominent problems summarized in the literature review (uneven infrastructure, low digital literacy, data security risks, and a lack of technical requirements). It also references related empirical literature on the drivers of digital elder care, selecting variables across four dimensions: technology, human resources, processes, and systems. The pain points revealed in the case study—such as “insufficient data literacy among nursing staff” and “the inability to directly link early warning information to medical systems”—were directly translated into core variables related to human factors (digital skills training for nursing staff) and technical factors (data platform interoperability), thereby effectively using situational evidence to support the selection of quantitative variables. Among these, the technology and process factors aim to address challenges such as “data silos” and “system interoperability”; the human resource factor focuses on the critical digital skills shortfall among caregivers; and the system factor considers the policy support conditions in the current pilot environment. Detailed definitions, measurement methods, and sources of specific variables are shown in Table 5.

**Table 5:** Analyzing results of digital elder care efficiency influencing factors based on the Tobit regression model.

Variable category	Influencing factors	Variable definition and measurement	Source and selection basis	Coefficient (Coef.)	Robust standard error (robust S.E.)	*Z*	*P*	Marginal effect (dy/dx)
Technical factors	Degree of Equipment Intelligent Integration	Scale Rating 1–5 points (1 = Low, 5 = High)	Literature review: responding to the challenges of low system integration in the case	0.042	0.015	2.80	0.005	0.028
	Data Platform Interoperability	Whether to integrate with the healthcare system (Yes = 1, No = 0)	Literature review, directly addressing the pain points of “data silos” and system interoperability.	0.067	0.023	2.91	0.004	0.045
	System Update and Maintenance Frequency	Average Annual Frequency (Times/Year)	Obtained from actual research, reflecting the sustainability of technical support and equipment availability.	0.031	0.011	2.82	0.005	0.021
	Degree of Device Brand Fragmentation	Number of Different Brand Devices Used (Units)	In-depth analysis of cases, reflecting negative friction costs caused by non-unified interfaces	−0.038	0.013	−2.92	0.004	−0.026
Human factors	Nursing Staff Digital Skills Training	Average Annual Training Duration (Hours)	Literature review, targeting the issues of “low digital literacy” and insufficient data interpretation skills	0.058	0.018	3.22	0.001	0.039
	Proportion of Digital Specialists	Dedicated Personnel/Total Personnel (%)	Organizational management theory, reflecting the organizational support strength for digital transformation	0.025	0.009	2.78	0.006	0.017
	Average Age of Staff	Years Old	Control variables: investigating the impact of the age structure of nursing teams on the acceptance of digital technology	−0.018	0.008	−2.25	0.024	−0.012
Process factors	Technology-Service Standardization Integration	Scale Rating 1–5 points (1 = Low, 5 = High)	Technology Empowerment Theory: Assess whether technology is truly embedded and optimizes service processes	0.052	0.017	3.06	0.002	0.035
	Incident Closed-Loop Resolution Rate	Number of successfully closed incidents/Total incidents (%)	Service Quality Theory: Reflects the quality of the service response loop from early warning to intervention	0.049	0.016	3.06	0.002	0.033
	Service Response Time	Average Response Time (minutes)	Actual Operating Data: Directly measure service efficiency and process smoothness	−0.036	0.014	−2.57	0.010	−0.024
Institutional factors	Scope of Medical Insurance Payment Coverage	Number of Covered Service Items (items)	Actual Operating Data: Directly measure service efficiency and process smoothness	0.022	0.010	2.20	0.028	0.015
	Data Security Certification Level	Scale Score 1–3 points (1 = Low, 3 = High)	Literature Review: Respond to the prominent issue of “insufficient data security and privacy protection.”	0.019	0.008	2.38	0.017	0.013
	Government subsidy intensity	Subsidy-to-investment ratio (%)	Current Status of Pilot Policies: Reflect the level of external institutional incentives in the early stages of transformation	0.016	0.007	2.29	0.022	0.011
Control variables	Institutional operating years	Year	Conventional Control Variables: Control for the impact of institutional experience accumulation on operational efficiency	0.012	0.006	2.00	0.046	0.008
	Average age of older adults served	Years old	Conventional Control Variables: Control for the overall disability level of service recipients and the difficulty of care	−0.014	0.006	−2.33	0.020	−0.009
	Regional Economic Level	Per Capita GDP Logarithm	Conventional Control Variables: Control for differences in macro-regional economies and resource endowments	0.021	0.009	2.33	0.020	0.014
Constant term	_cons			0.452	0.121	3.74	0.000	

Further analysis of the factors influencing the efficiency values using the Tobit regression model revealed (see Table 5):

Positive significant factors: frequency of training in digital skills for nursing staff (*p* < 0.01) and the degree of standardized integration of technology and service processes (*p* < 0.05).

Negative significant factors: degree of fragmentation of equipment brands and data interfaces (*p* < 0.01).

Coverage of medical insurance payment policies at the institutional level did not have a significant impact in this pilot phase.

### 5.4 Quantitative analysis of cost control effects

The comparative analysis shows (see Table 6) that in units that achieve DEA efficiency, the application of digital technology has led to significant optimization of the cost structures:

**Table 6:** Quantitative analysis of the cost control effects of digital technology that empowers healthy older adults.

Cost category	Cost breakdown items	Annual Average cost of traditional model (CNY 10,000)	Annual average cost of digital empowerment model (CNY 10,000)	Absolute cost savings (CNY 10,000)	Relative cost savings rate	Cost-benefit realization period	Key quantifiable metrics
1. Direct operating costs	Labor Costs						
	Routine Inspection and Care Labor Costs	18.5	9.8	8.7	47.0%	Instant	Patrol frequency reduced 60%
	Emergency incident response labor cost	6.2	3.5	2.7	43.5%	Instant	Response time reduced 58%
	Health record management fee	3.8	1.2	2.6	68.4%	Instant	Automation rate: 85%
	Subtotal	28.5	14.5	14.0	49.1%		
	Equipment and Consumables Cost						
	Traditional Care Equipment Procurement and Maintenance	5.6	3.2	2.4	42.9%	1–2 Years	Equipment Reuse Rate Improvement35%
	Disposable medical consumables	8.9	6.1	2.8	31.5%	Instant	Accurate demand forecasting reduces waste.
	Smart device depreciation and maintenance	0.0	7.5	−7.5	—	1–3 years	High initial investment
	Subtotal	14.5	16.8	−2.3	−15.9%		
2. Indirect medical costs	Preventive cost savings						
	Avoidable emergency room costs	12.3	4.9	7.4	60.2%	6–12months	Early warning effectiveness rate: 72%
	Avoidable hospitalization costs	25.8	18.6	7.2	27.9%	12–24months	Hospitalization rate reduction: 23%
	Complication management costs	9.7	6.3	3.4	35.1%	6–18months	Chronic disease control achievement rate improved 41%
	Subtotal	47.8	29.8	18.0	37.7%		
3. Opportunity and risk costs	Efficiency Improvement Benefits						
	Value per unit time of nursing staff	—	5.2	5.2	—	Instant	Productivity Improvement: 25%
	Benefits from Increased Service Coverage	—	8.7	8.7	—	6–12months	Increase in Number of People Served: 40%
	Risk Avoidance Value						
	Reduction in Safety Accident Compensation	3.5	0.8	2.7	77.1%	Instant	Decrease in Falls and Other Incidents 65%
	Reduced Legal Litigation Costs	1.2	0.3	0.9	75.0%	Instant	Decrease in Dispute Rate: 70%
	Subtotal	4.7	15.0	10.3	219.1%		
4. Comprehensive cost analysis	Annual Total Cost	95.5	76.1	19.4	20.3%		
	Initial Technology Investment Allocation	0.0	12.3	−12.3	—	3–5 Years Depreciation	Investment Payback Period: 2.8 Years
	Net Cost-Benefit	95.5	88.4	7.1	7.4%		
	Cost-Benefit Ratio (CBR)	1.00	1.32	0.32	—		For every 1 yuan invested, 1.32 yuan in benefits are generated.

Preventive Cost Benefits: By early warning and transforming some health events that originally required emergency hospitalization into community or home-based early intervention, it is estimated that approximately 15%–20% of subsequent high medical expenses can be reduced.

Human resource cost benefits. Automated monitoring and alarming partially free nursing staff from frequent routine patrols, allowing them to focus more on high-value services that require human judgment and emotional communication, resulting in an approximately 25% increase in unit labor efficiency.

The “efficiency-cost” paradox: Initial technological investments lead to higher current-period costs (as shown in Table 6, depreciation and maintenance of smart devices add 75,000 yuan to current-period equipment costs, resulting in a 15.9% increase in equipment-related costs); however, after a 1–2-year operational cycle, the resulting efficiency gains and risk mitigation begin to offset and eventually exceed the initial investment. This conclusion is supported by actual operational data from the pilot program in City A, which has been running for over 18 months, as well as full-lifecycle project calculations: In terms of the cost-benefit realization cycle, the primary risk mitigation effects—such as avoided hospitalization costs (savings of 72,000 yuan) and complication management costs (savings of 34,000 yuan)—are concentrated within the first 12 to 24 months; combined with immediate savings on labor costs (140,000 yuan), the total annual cost savings (194,000 yuan) gradually offset the amortized initial technology investment (123,000 yuan) within 1–2 years. The comprehensive payback period is 2.8 years, yielding a net cost-benefit of 71,000 yuan (cost-benefit ratio of 1:1.32), thereby validating the aforementioned “J-curve” effect from a cross-cycle perspective.

Quantitative analysis of cost-control effects (see Table 6) shows that digital technology supporting healthy aging has produced significant cost restructuring. In a standard unit serving 100 older adults, the annual total cost savings rate reached 20.3%, with the greatest savings in human resource costs (49.1%). However, dynamic changes in the cost structure should be noted: initial investment in smart devices will increase current equipment costs by 15.9%, forming a “J-curve effect,” meaning short-term cost increases and long-term cost decreases. The “preventive savings” in medical costs have a clear leverage effect, accounting for 36.1% of total savings, reflecting the economic logic of “prevention over cure” in health management. The calculated investment payback period is 2.8 years, and the cost-benefit ratio (CBR) is 1:1.32, indicating strong economic feasibility for the digital technology investment. This cost-control effect is scale-sensitive: when the service scale expands from 50 to 500 people, the unit cost can be reduced by 41.4%, highlighting the importance of scaled operations.

## 6 Prospective transformation paths: trend prediction based on blockchain and AI

Although digital technology for empowering healthy aging has improved service efficiency and demand matching, core pain points remain, including insufficient data security, insufficient personalization, unbalanced resource allocation, and a disconnect between technology and care services (case-pilot issues). Based on this, by combining core theories such as technology empowerment theory, resource allocation theory, and data security theory, and looking ahead to the deep application trends of blockchain and AI technologies, we construct a deepened digital transformation path that combines empirical support with theoretical depth. This serves both as a response to the empirical results presented earlier and as a supplement and extension of the theoretical system of digital technology that empowers healthy aging.

### 6.1 Blockchain technology: transformation of health and elder care data governance based on data security theory

Currently, the field of digital health and older adult care faces significant issues, including non-standardized data collection, insecure storage and transmission, and imperfect sharing mechanisms, which pose a high risk of health data privacy breaches. Simultaneously, data fragmentation across entities (medical institutions, older adult care institutions, and nursing enterprises) impedes further improvements in service efficiency. Combining data security theory and resource allocation theory, the core characteristics of blockchain technology, such as decentralization, immutability, traceability, and encrypted sharing, can precisely address these pain points, promote the transformation of the health and older adult care data governance model, and provide sustainable security support for digital technology empowerment (32, 33).

Based on the results of the SPSS multiple-choice analysis in the previous text, the application of blockchain technology should consider the diverse needs of different older adult care groups: for the home older adult care group, the focus should be on building a blockchain notarization system of personal health data to ensure the authenticity and security of remote consultation and health monitoring data, thereby improving the precision of home older adult care services; for the institutional older adult care group, an inter-institutional blockchain data sharing platform should be established to achieve interoperability of nursing and rehabilitation data, addressing the problem of “disconnection between nursing services and ADL monitoring data” mentioned in the previous case, and further improving the overall efficiency of institutional older adult care (promoting the improvement of DEA pure technical efficiency); for the rural older adult care group, lightweight blockchain technology should be used to reduce data storage and transmission costs, solve the data sharing challenges caused by weak digital infrastructure in rural areas, promote the extension of digital health and older adult care services to rural areas, and alleviate the problem of “uneven application of digital infrastructure” raised earlier (34, 35).

### 6.2 AI technology: personalized service transformation based on technology empowerment and service quality theory

In the empirical analysis presented earlier, the SPSS multiple-choice analysis clearly showed significant differences in digital service demand between different older adult care groups (Chi-square test, *P* < 0.05): the home care group focused on remote consultation and health monitoring, the institutional care group emphasized smart nursing, and the community care group prioritized emergency rescue and dissemination of health knowledge. Meanwhile, the DEA efficiency evaluation indicated that although the scale efficiency of digital group institutions had improved, the lack of personalized services led to only a limited improvement in service quality. This aligns perfectly with the central issue of “disconnection between technology application and older adult needs” raised previously. Combining technology empowerment theory and service quality theory, the deep application of AI technologies (machine learning, natural language processing, computer vision) can achieve a transformation of health and older adult care services from “generic” to “personalized,” addressing the pain point of the demand-service mismatch, further improving service efficiency and quality, and promoting the refined development of digital technology empowerment in health and older adult care (36, 37).

Specifically, the transformation to personalized services based on AI technology must closely integrate the empirical results mentioned above, focusing on the differing needs of various older adult groups, and establish a stratified and categorized personalized service system: First, based on machine learning technology, integrate the demand data of 1,086 older adults collected through the above questionnaire survey, ADL monitoring data (case pilot data), and health data to construct a demand profile model for the older adults, accurately identifying the core needs of older adults of different ages, health conditions, and caregiving models. The cross-analysis of the previously mentioned SPSS multiple-choice analysis has preliminarily revealed this heterogeneity (38, 39). In terms of age, the requirements for “emergency calls and safety monitoring” among older adults aged 85 and above account for as much as 58.3%, significantly higher than other age groups, while the requirements for “online entertainment and social interaction” among younger older adults (aged 60–74) reach 44.1%; regarding health status, older adults with one or more chronic diseases (accounting for 79.9% of the sample) have requirements for “remote medication guidance and consultation” that reach 65.4%. Based on the above data characteristics, the AI model can implement precise differentiated interventions: for the older adults with high disability, relying on computer vision technology and ADL monitoring data to achieve accurate warnings of risks such as falls and abnormal stillness, while providing personalized assistance services for dressing, washing, etc., through AI nursing robots, addressing the conclusion in the case that “there is still room for improvement in the nursing outcomes for disabled older adults individuals” (40); adults individuals with chronic diseases, based on machine learning algorithms, analyze their vital signs data and lifestyle data to formulate personalized health intervention plans (diet, exercise, medication reminders) to reduce the incidence of disease, responding to the empirical result that “the effectiveness of health risk interventions need enhancement” (41); for younger adults individuals, relying on natural language processing technology, build an AI health consultation platform to provide personalized health knowledge dissemination and wellness advice to meet their health needs (42).

## 7 Conclusions

### 7.1 Main research conclusions

Digital technology has a significant positive effect on improving the efficiency of healthcare and older adult care services, with an average comprehensive technical efficiency of 0.845, where management capability and technology integration are key. Technology empowerment exhibits an efficiency-cost-time-lag effect that, in the long term, can achieve cost control by improving human efficiency and preventing disease progression. The needs of older adults are highly heterogeneous, requiring precise profiling using methods such as multiple-choice analysis to avoid a “one-size-fits-all” approach to technology supply.

The current transformation faces bottlenecks such as data security, silo effects, and a lack of personalization. Blockchain and AI are key technological directions for overcoming these bottlenecks and advancing empowerment. Successful transformation relies on deep synergy among technology, business models, and policy systems.

### 7.2 Policy recommendations to optimize the efficiency of digital technology empowerment

The existing literature has proposed macro-level transformation pathways, such as strengthening infrastructure construction and improving data security systems (these consensus-based suggestions provide the legitimacy basis for the policy in this document). Still, most lack empirical support and quantifiable benchmarks, leading to suggestions remaining at the level of “ should” rather than being actionable. The core contribution of this research lies in transforming macro consensus into precise, quantifiable “actual” measures based on DEA efficiency measurement, Tobit regression of influencers, and quantitative analysis of cost control, thereby clarifying the priority and intensity of policy intervention. The specific policy recommendations are as follows:

First, develop data standards and interoperability specifications to break the efficiency losses caused by “islands.” The earlier Tobit regression indicates that equipment brand fragmentation has a significant negative impact on efficiency (*p* < 0.01), and case pilot projects have revealed an inability to link ADL monitoring data to medical systems directly. Therefore, it is recommended to introduce Digital Health Older Adults Care Equipment Data Interface Standards, which require newly purchased equipment and platforms to comply with unified data interoperability standards; at the same time, establish special subsidy incentives for existing institutions to carry out interface transformations, breaking down data barriers between health monitoring, electronic medical records, and nursing task orders, and converting data interoperability into increased scale efficiency.

Second, promote the training and certification of composite talents in “digital skills + nursing skills” to compensate for losses in pure technical efficiency. DEA measurement shows that the average pure technical efficiency (PTE) is 0.901, indicating that technical inefficiency is the main source of inefficiency. At the same time, a Tobit regression confirms that digital skills training for nursing staff has a significant positive impact on efficiency (*p* < 0.01). It is recommended that the human resources department take the lead in formulating certification standards for “Digital Older Adults Care Workers,” incorporating smart device operation and ADL data anomaly judgment into mandatory modules for vocational training and qualification assessment systems; in response to the current negative impact of the average age of personnel on efficiency, institutions are advised to establish team models that combine “mentoring” and “digital specialists” roles to enhance overall data literacy.

Third, explore “value-based medical insurance payment” and dynamic pricing mechanisms to smooth the “J-curve” cost pains. Cost analysis indicates that digital technology investments have a 2.8-year payback period and exhibit a “J-curve” effect, in which rising initial equipment costs of 15.9% constrain institutions' willingness to transform. It is recommended that the medical insurance bureau gradually include effective digital services such as preventive monitoring (e.g., ADL anomaly warnings) and remote interventions within the scope of medical insurance payments, and based on the calculated preventive cost savings rate (37.7%), explore a “value-based payment” mechanism to leverage medical insurance to offset initial costs for institutions and incentivize closed-loop services that emphasize “prevention over treatment”.

Fourth, encourage a “racing mechanism” and pilot projects to guide the expansion of units with increasing returns to scale in service coverage. DEA results show that 55% of units exhibit increasing returns to scale (primarily in community and home settings), and cost analysis indicates that unit costs can be reduced by 41.4% as service scale expands from 50 to 500 people. It is recommended that the government establish a Digital Older Adults Care Scalable Operations Pilot Fund to support pilot competitions for different technological paths (such as various blockchain solutions) within manageable scopes; for community/home service units that achieve DEA efficiency and are in a state of increasing scale returns, priority should be given to bed subsidies or service procurement tilts to promote cross-regional replication and scalable operations.

Fifth, strengthen specifications for older-adult-friendly design and privacy and security certification to eliminate supply-demand mismatches caused by demand heterogeneity. In light of the significant demand differences shown by older adults in multiple option analyses and a 15% false positive rate and resistance in case pilot projects, it is recommended to introduce “Mandatory National Standards for the Design of Older Adults-Friendly Smart Devices” to regulate interaction logic and comfort of wearing, thus reducing false positive rates; at the same time, establish a tiered certification system for the security of digital older adults care privacy, implementing data minimization and desensitization for different groups (e.g., emergency calls for people of high age living alone, remote consultations for patients with chronic diseases) to alleviate the privacy anxiety of older adults about being “monitored” and improve user satisfaction (Y3).

### 7.3 Research limitations and future prospects

This initial DEA measurement only used 20 groups of samples, and through data supplementation from cross-district pilot projects during the revision phase, the DMUs were expanded to 52, addressing the calculation bias caused by small samples; the study relies mainly on cross-sectional data and specific pilot projects, and future longitudinal tracking studies could observe dynamic changes in efficiency. There is still room to optimize the indicator selection for the DEA model. At the methodological level, this study adopts a balanced approach, explicitly defining the research design as primarily quantitative DEA/Tobit analysis, supplemented by evidence from contextual case studies. Qualitative case materials are primarily used to provide empirical grounding for the construction of quantitative indicators and the interpretation of results, rather than being analyzed as independent qualitative leads. Future research could further incorporate standard qualitative data collection and analysis procedures, such as semi-structured interviews and focus groups, and adopt classic mixed-methods designs (such as exploratory sequential designs) to deepen the qualitative deconstruction of the mechanisms through which digital technologies empower individuals. As blockchain and AI applications deepen, evaluating their impact on efficiency and fairness will be an important next research topic. The digital transformation of health and care for older adults is a profound revolution that requires continuous exploration and practice across industry, academia, research, government, and users.

## Data Availability

The original contributions presented in the study are included in the article/Supplementary Material, further inquiries can be directed to the corresponding author/s.
